# Immune Suppressive Effects of Tonsil-Derived Mesenchymal Stem Cells on Mouse Bone-Marrow-Derived Dendritic Cells

**DOI:** 10.1155/2015/106540

**Published:** 2015-02-16

**Authors:** Minhwa Park, Yu-Hee Kim, Jung-Hwa Ryu, So-Youn Woo, Kyung-Ha Ryu

**Affiliations:** ^1^Department of Microbiology, School of Medicine, Ewha Womans University, Seoul 158-710, Republic of Korea; ^2^Department of Pediatrics, School of Medicine, Ewha Womans University, Seoul 158-710, Republic of Korea

## Abstract

Mesenchymal stem cells (MSCs) are considered valuable sources for cell therapy because of their immune regulatory function. Here, we investigated the effects of tonsil-derived MSCs (T-MSCs) on the differentiation, maturation, and function of dendritic cells (DCs). We examined the effect of T-MSCs on differentiation and maturation of bone-marrow- (BM-) derived monocytes into DCs and we found suppressive effect of T-MSCs on DCs via direct contact as well as soluble mediators. Moreover, T cell proliferation, normally increased in the presence of DCs, was inhibited by T-MSCs. Differentiation of CD4^+^ T cell subsets by the DC-T cell interaction also was inhibited by T-MSCs. The soluble mediators suppressed by T-MSCs were granulocyte-macrophage colony-stimulating factor (GM-CSF), RANTES, interleukin-6 (IL-6), and monocyte chemoattractant protein-1 (MCP-1). Taken together, T-MSCs exert immune modulatory function via suppression of the differentiation, maturation, and function of BM-derived DCs. Our data suggests that T-MSCs could be used as a novel source of stem cell therapy as immune modulators.

## 1. Introduction

Mesenchymal stromal cells (MSCs) have known regulatory effects on immune and inflammatory responses [1]. Furthermore, bone-marrow-derived MSCs (BM-MSCs) regulate the functions of immune cells such as T cells [2–4], dendritic cells (DCs), B cells, and natural killer cells [5]. Among these, DCs are the orchestrators of the immune response because of their function as antigen-presenting cells. We previously showed that BM-MSCs can inhibit DC maturation and motility [6]. Thus, because MSCs participate in the immune modulatory function of DCs, they might be useful as cell therapy agents for various autoimmune diseases. Although MSCs have a known immune modulatory effect on DC maturation and migration, their influence on antigen presentation function and recruitment of DCs has not been studied.

In a previous report, we confirmed that tonsil-derived stromal cells (T-MSCs) have characteristics of MSCs [7]. In comparison with BM-MSCs, adipose-derived (AD) MSCs have similar immunomodulatory effects [8]. Although AD-MSCs reduced inflammatory and T cell responses via interleukin-10 (IL-10) secretion and induction of T_reg_ cells [9], the immunomodulatory effects of T-MSCs have not been characterized. Therefore, in this study we evaluated the immunomodulatory effects of T-MSCs on DC and characterized their mechanism of action.

## 2. Materials and Methods

### 2.1. Mice

Eight-week-old female Balb/c mice (OrientBio, Korea) were used. All procedures and protocols were approved by Ewha Womans University College of Medicine Animal Ethics Committee (ESM 11-0222). Mice were kept at 21°C~23°C and 51%~54% humidity with a 12 h light/dark cycle. Food and water were available ad libitum.

### 2.2. Harvest and Culture of T-MSCs

Tonsils were obtained with informed consent from patients undergoing tonsillectomy and Institutional Review Board approval (ECT 11-53-02, Ewha Womans University, Mok-Dong Hospital, Seoul, Korea). Fresh palatine tonsils were washed five times with PBS, followed by mincing with blade and digested in RPMI 1640 medium containing 210 U/mL collagenase type I (Invitrogen) and 10 *μ*g/mL DNase (Sigma-Aldrich, St. Louis, MO, USA) for 30 min at 37°C. Following filtration through a cell strainer, the cells were washed twice in 20% normal human serum (PAA Laboratories GmbH, Austria) containing RPMI 1640 medium and once with 10% human serum/RPMI 1640. Mononuclear cells were obtained from cell suspension by Ficoll-Paque (GE Healthcare, Buckinghamshire, UK) density gradient centrifugation. The cells were plated at a density of 1 × 10^7^ cells per 100 mm diameter in culture dishes in DMEM containing 10% fetal bovine serum (FBS) with antibiotics. After 24 h, nonadherent cells were removed by pipetting and replenished with RPMI 1640 containing antibiotics and 10% FBS. For the following experiment, T-MSCs (passage 4) were precultured for 24 hrs at 5 × 10^5^ cells/well in a 6-well plate in RPMI 1640 supplemented with 10% heat-inactivated fetal bovine serum (FBS; Welgene, Korea) and 1% penicillin/streptomycin [7].

### 2.3. Generation and Differentiation of BM-DCs

Female Balb/c mice were sacrificed and BM was obtained from both femur and tibia. Femurs and tibias were flushed with 5 mL RPMI 1640, and red blood cells (RBCs) were lysed using ACK buffer (10x, 1.5 M NH_4_Cl, 100 mM KHCO_3_, and 10 mM disodium EDTA). Cell pellets were resuspended and harvested at each well 2 × 10^6^ cells in 6-well cell culture plate with RPMI 1640 containing 10% FBS and 1% penicillin/streptomycin. DCs were induced to differentiate by the addition of recombinant mouse GM-CSF (rmGM-CSF, R&D Systems, Minneapolis, MN, USA) (20 ng/mL) every 3 days and lipopolysaccharide (LPS) (1 *μ*L/mL, Sigma, St. Louis, MO, USA) on day 10. After another 48 hrs, cells and medium were collected for further assays.

### 2.4. Coculture of BM-DCs with T-MSCs

In order to prevent contact between BM-DCs and T-MSCs, we used a transwell plate with a 0.4 *μ*m pore size polycarbonate membrane (Corning, Acton, MA). BM-DCs and T-MSCs were placed in the upper and lower chambers of the plate (ratio 5 : 1), respectively, and were cultured in RPMI 1640 medium with 10% FBS, 1% penicillin, and 20 ng/mL rmGM-CSF. LPS (1 *μ*L/mL) was added to the upper chamber on day 10 to induce maturation of BM-DCs.

### 2.5. Flow Cytometry

For phenotyping, cells were incubated with the following fluorochrome-conjugated antibodies at final concentration of 2 ug/mL: FITC-anti-mouse CD11b (M1/70, Rat IgG_2b_, eBiosciences, San Diego, CA), PE-anti-mouse CD11c (HL3, Hamster IgG_1_, BD Biosciences, Franklin Lakes, NJ), APC-anti-mouse CD11c (N418, Hamster IgG, BioLegend, San Diego, CA), PerCP-anti-mouse CD80 (16-1 OA1, Hamster IgG_2_, BD Biosciences), PE-anti-mouse CD86 (GL1, Rat IgG_2a_, BD Biosciences), PE-anti-mouse CD14 (rmC5-3, Rat IgG_1_, BD Biosciences), PE-anti-mouse class II major histocompatibility complex (MHC) (2G9, Rat IgG_2a_, BD Biosciences), and PE-anti-mouse CCR7 (4B12, Rat IgG_2a_, R&D Systems). For controls, no stained cells were used. Cells were centrifuged at 300 ×g for 5 min at RT, washed, and fixed in 1% paraformaldehyde in phosphate buffered saline (PBS).

For intracellular staining, cells were fixed in 4% paraformaldehyde (PFA) in PBS for 20 min on ice and then washed with 20% permeabilization buffer (0.5% saponin, 1% bovine serum albumin (BSA) in PBS) plus 0.5% FBS in PBS. Cells were resuspended and incubated with the following fluorochrome-conjugated antibodies for 30 min on ice: FITC-anti-mouse CD4 (RM4.5, Rat IgG_2a_, BD Biosciences), APC-anti-mouse interferon gamma (IFN-*γ*) (XMG1.2, Rat IgG_1_, BioLegend), PE-anti-mouse interleukin-4 (IL-4) (11B11, Rat IgG_1_, BD Biosciences), and PE-anti-mouse interleukin-17A (IL-17A) (TC11-18H10.1, Rat IgG_1_, BioLegend). Following staining, cells were washed, resuspended in 0.5% FBS in PBS, and analyzed by flow cytometry with Cell Quest software (BD Biosciences).

### 2.6. Isolation of CD4^+^ T Cells

Spleens were obtained from 8-week-old female Balb/c mice and were manually disrupted in 5 mL RPMI1640 medium. Cell suspensions were washed and resuspended with 600 *μ*L MACS sorting buffer (containing 0.5% BSA, 2 mM EDTA in PBS, pH 7.2) followed by addition of anti-CD4 or anti-CD3 microbead solution (10 *μ*L per 10^7^ cells). Cells were incubated on ice for 15 min on ice, washed, and resuspended in 1 mL sorting buffer. Cells were separated using a MACS magnetic column (Miltenyi Biotec, Auburn, CA, USA) according to the manufacturer's protocol. Separated cells were stained with PE-conjugated anti-mouse CD4 antibody (GK 1.5, Rat IgG_2b_, BioLegend) and analyzed by flow cytometry to determine the purity of the cell population.

### 2.7. T Cell Proliferation and CD4^+^ T Subset Differentiation

To measure proliferation, 2 × 10^8^ cells were stained with 5 *μ*M carboxyfluorescein succinimidyl ester (CFSE, Invitrogen, Carlsbad, CA, USA) for 5 min at room temperature (RT); then 1 mL FBS was added to stop the reaction. Cells were washed three times in RPMI 1640 with 10% FBS and transferred to a 6-well culture plate (2 × 10^6^ cells/well). T-MSCs or BM-DCs were added and cultures were incubated for 48 hrs. For T cell stimulation, 5 ug/mL each of LEAF purified anti-mouse CD3 (17A2, Rat IgG_2b_, BioLegend) and LEAF purified anti-mouse CD28 (37.51, Syrian Hamster IgG, BioLegend) antibodies were diluted in RPMI 1640 with 10% FBS and antibiotics and added to the cells. Cell numbers are 4 × 10^5^ cells/well for T-MSCs, for 2 × 10^6^ cells/well BM-DCs, and for 2 × 10^6^ cells/well T cells. After 48 hrs, cells were collected for flow cytometric analysis and supernatants were assayed by Cytokine Array.

### 2.8. Cytokine Array

Collected culture medium and Human Cytokine Array C1 Kit (RayBiotech, Norcross, GA, USA) components were equilibrated to RT and the assay was performed according to the manufacturer's instructions. The developed membrane was analyzed by a chemiluminescence imaging system (LAS-3000, Fujifilm, Japan).

### 2.9. Statistical Analysis

Data are expressed as the mean ± standard error of the mean (SEM). One-way or two-way ANOVA was used for group analysis, and Student's *t*-test was used to identify statistically significant differences in staining (at *P* < 0.05). Statistical analyses were performed using GraphPad Prism Software (GraphPad Software Inc., San Diego, CA).

## 3. Results

### 3.1. T-MSCs Inhibited Differentiation of CD11b^+^ DCs from BMCs under Coculture Conditions

**Figure 1:**
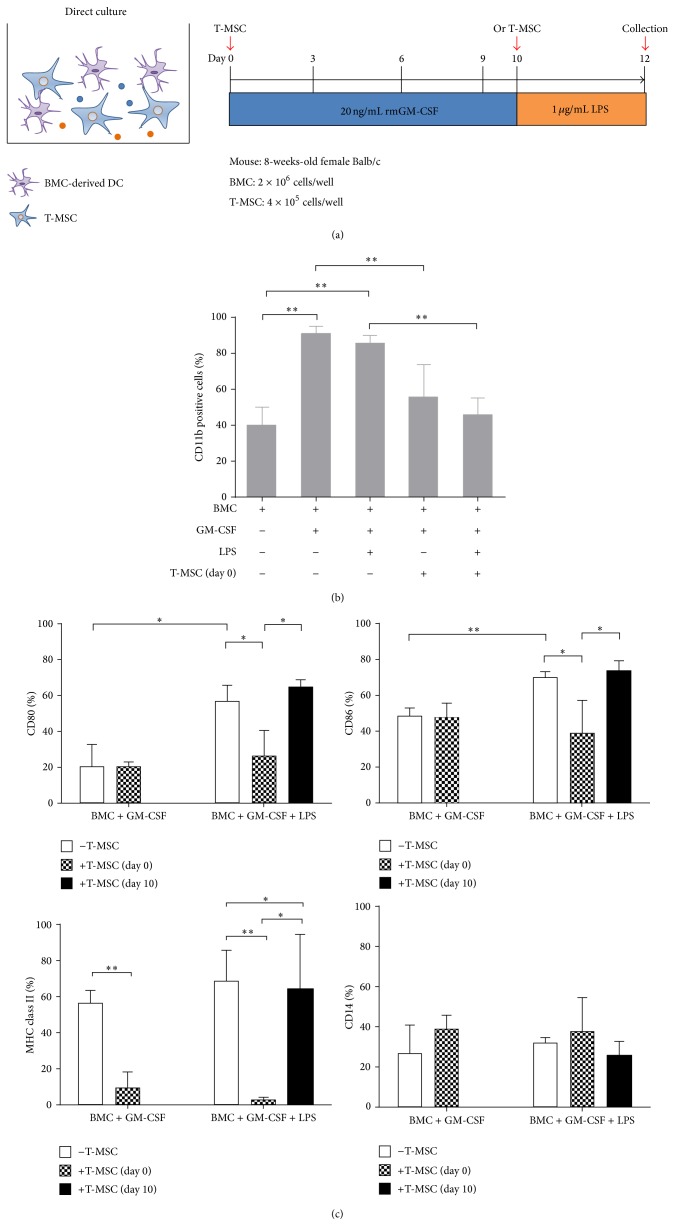
T-MSCs inhibit differentiation and maturation of DCs under coculture conditions. Bone-marrow- (BM-) derived monocytes (BMCs) isolated from 8-week-old BALB/c mice were incubated with of granulocyte-macrophage colony-stimulating factor (GM-CSF) (20 ng/mL) for 12 days to induce differentiation into dendritic cells (DCs). (a) Lipopolysaccharide (LPS) (1 *μ*g/mL) was added for the last 2 days to induce DC maturation. (b) GM-CSF treatment induced expansion of CD11b^+^ cells (^**^ *P* < 0.01). (c) Tonsil-derived MSCs (T-MSCs) added at day 0, but not at day 10, inhibited upregulated expression of CD80 (^*^ *P* < 0.05) and CD86 (^**^ *P* < 0.01) on mature DCs by direct contact. Upregulated major histocompatibility complex (MHC) class II expression on immature (^**^ *P* < 0.01) and mature DCs (^**^ *P* < 0.01) was reduced by T-MSCs, but CD14 expression was not affected.

Mouse BM cells (BMCs) were cultured with rmGM-CSF (20 ng/mL) for 10 days to induce differentiation into immature DCs, followed by the addition of LPS for 48 hrs to induce DC maturation. T-MSCs (4 × 10^5^ cells/well) were added to culture plates providing cell-to-cell contact on either day 0 or day 10 of BMC culture (Figure 1(a)). The proportion of CD11b^+^ BMCs was markedly increased from 38.9 ± 4.8% to 90.7 ± 1.9% (*P* < 0.05) with the addition of GM-CSF (20 ng/mL) with or without LPS for 10 days (Figure 1(b)). However, the addition of T-MSCs on day 0 inhibited CD11b^+^ cell expansion (55.1 ± 8.3%), and this effect was not affected by the addition of LPS (57.7 ± 8.9%). Thus, T-MSCs inhibited differentiation of BMC-derived DCs by GM-CSF under cell-to-cell contact conditions (Figure 1(b)).

LPS stimulation increased the expression of the costimulatory molecules CD80 (56.8 ± 5.2%), CD86 (69.7 ± 3.4%), and class II MHC (64.5 ± 17.31%) on DCs; however, the proportion of CD14^+^ cells did not change. In contrast, the addition of T-MSCs from day 0 inhibited the upregulated expression of CD80 and CD86 induced by LPS but did not affect class II MHC expression. In addition, when T-MSCs were added from day 10, there was no inhibitory effect on the expression of the costimulatory molecules (Figure 1(c)). Therefore, T-MSCs likely inhibit BM-DC maturation as evidenced by the reduced expression of costimulatory molecules and class II MHC under cell contact conditions.

### 3.2. T-MSCs Inhibited Differentiation and Maturation of CD11b^+^ DCs from BMCs under Transwell Conditions

**Figure 2:**
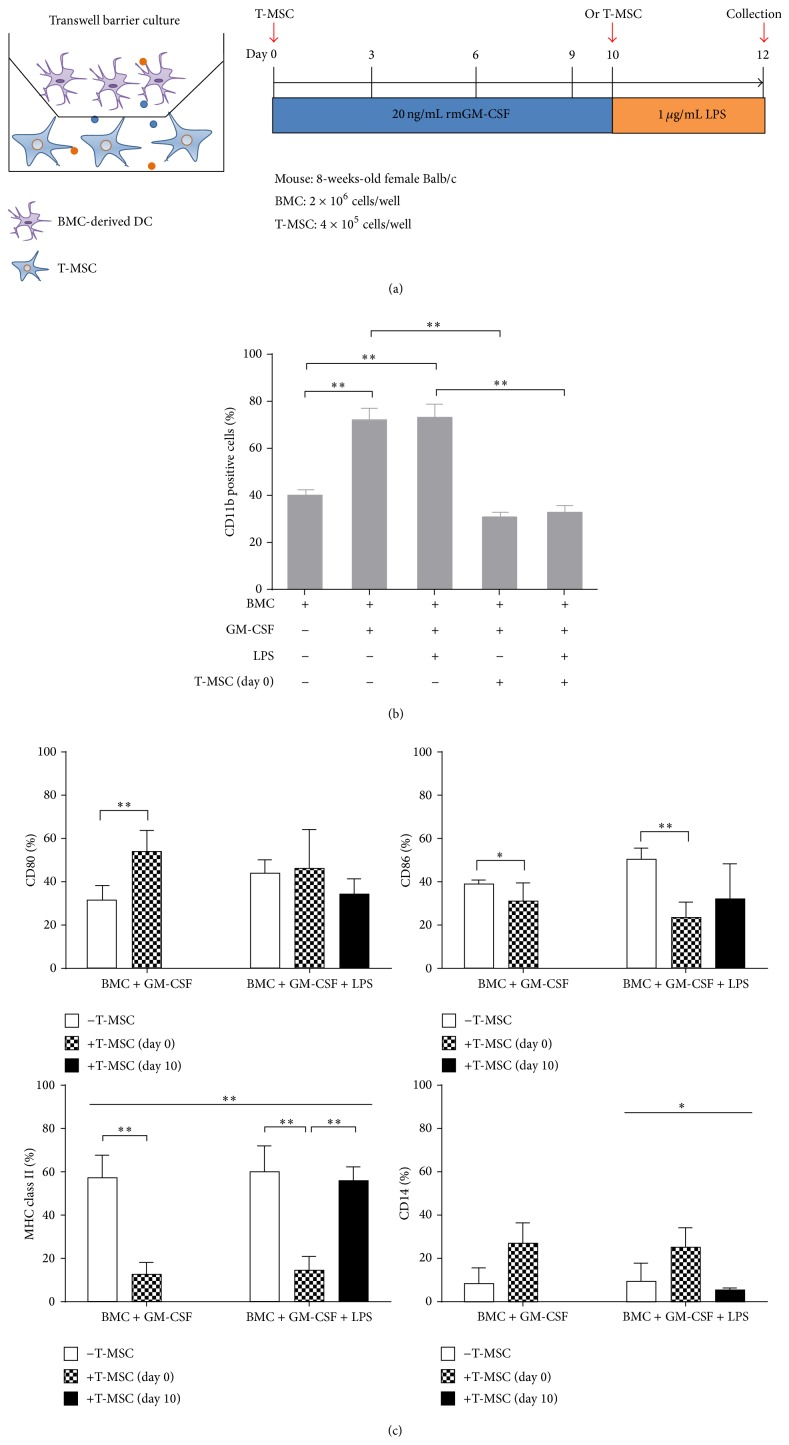
T-MSCs inhibit differentiation and maturation of DCs by secretion of soluble factors. (a) BMCs and T-MSCs were cultured in a transwell plate and BMCs were induced to differentiate into DCs by GM-CSF. (b) BMCs differentiation into CD11b^+^ cells was inhibited by T-MSCs added at day 0 (^**^ *P* < 0.01). (c) CD86 expression on mature DCs was significantly downregulated by T-MSCs (^**^ *P* < 0.01). Similar to the coculture condition, MHC class II expression on immature and mature DCs was inhibited by factors secreted from T-MSCs (^**^ *P* < 0.01). CD14 expression by T-MSCs showed a trend toward increase, but it was not significant.

Using the transwell culture plate, we investigated whether the ability of T-MSCs to inhibit DC differentiation required cell contact or was mediated through soluble factors (Figure 2(a)). T-MSCs inhibited the expansion of CD11b^+^ cells in the BM-DCs induced by GM-CSF, with or without LPS stimulation (Figure 2(b)). With the addition of T-MSCs from day 0, expression of CD86 and class II MHC was decreased as well, irrespective of LPS stimulation (Figure 2(c)).

### 3.3. T-MSCs Inhibited DC-Mediated T Cell Proliferation and CD4^+^ T Cell Differentiation

**Figure 3:**
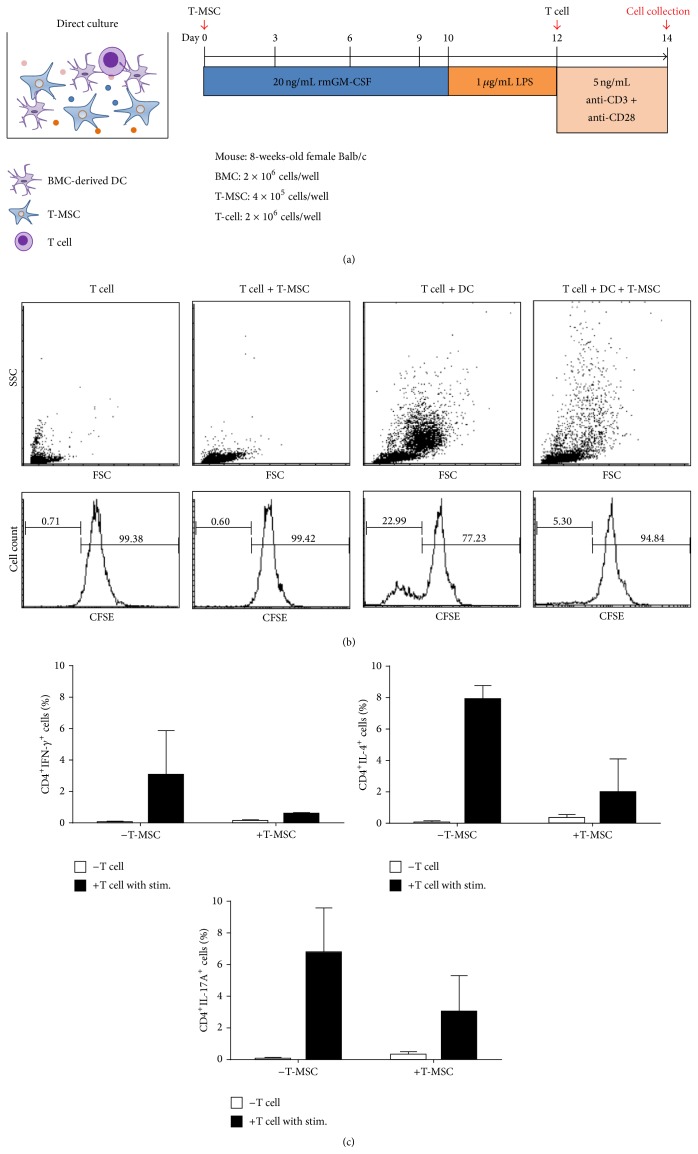
T-MSCs inhibit DC-mediated T cell proliferation and differentiation of the CD4^+^ T cell subset. (a) T cells were isolated from mouse spleens (BALB/c, 8-week-old, female) using MACS. (b) T cells were cocultured with mature DCs in the presence or absence of T-MSCs, and differentiation into CD4^+^ T cell subsets was induced using anti-CD3 and anti-CD28 antibodies. T cell numbers were increased upon coculturing with mature DCs. In the presence of T-MSCs, however, T cell proliferation was inhibited by 80%. (c) Differentiation into Th1, Th2, and Th17 cells also was decreased upon addition of T-MSCs.

In order to determine the effect of T-MSCs cocultured with BM-DCs on T cell proliferation, we isolated T cells from mouse spleens and labeled them with CFSE to measure cell division. As a control group, we also cultured CD4^+^ T cells with T-MSCs or with BM-DCs alone (Figure 3(a)). Proliferating T cells, represented as peaks of decreasing CFSE intensity, were reduced from 22.99% to 5.3% after the addition of T-MSCs to the culture (Figure 3(b)). Therefore, 22.99% of CD4^+^ T cells cultured with BM-DCs and stimulated with anti-CD3/anti-CD28 divided once, but upon coculture with T-MSCs from day 0, cell division was decreased to 5.30% (Figure 3(b)).

To evaluate the differentiating capacity of CD4^+^ T cell with BM-DCs, we isolated CD4^+^ T cells from mouse spleen and cocultured them with BM-DCs and anti-CD3/anti-CD28 stimulation, with or without T-MSCs. After 48 hrs, we collected the cells and stained for flow cytometry analysis. We found that the percentage of CD4^+^INF-*γ* ^+^ (T_H_1 cell), CD4^+^IL-4^+^ (T_H_2 cell), and CD4^+^IL17A^+^ (T_H_17) cells was increased after coculture with BM-DCs and stimulation with anti-CD3/anti-CD28 antibodies. However, when cocultured with T-MSC-preconditioned BM-DCs, the proportions of differentiated T cells were reduced (Figure 3(c)).

### 3.4. T-MSCs Modulate Cytokine Secretion from Mature DCs

**Figure 4:**
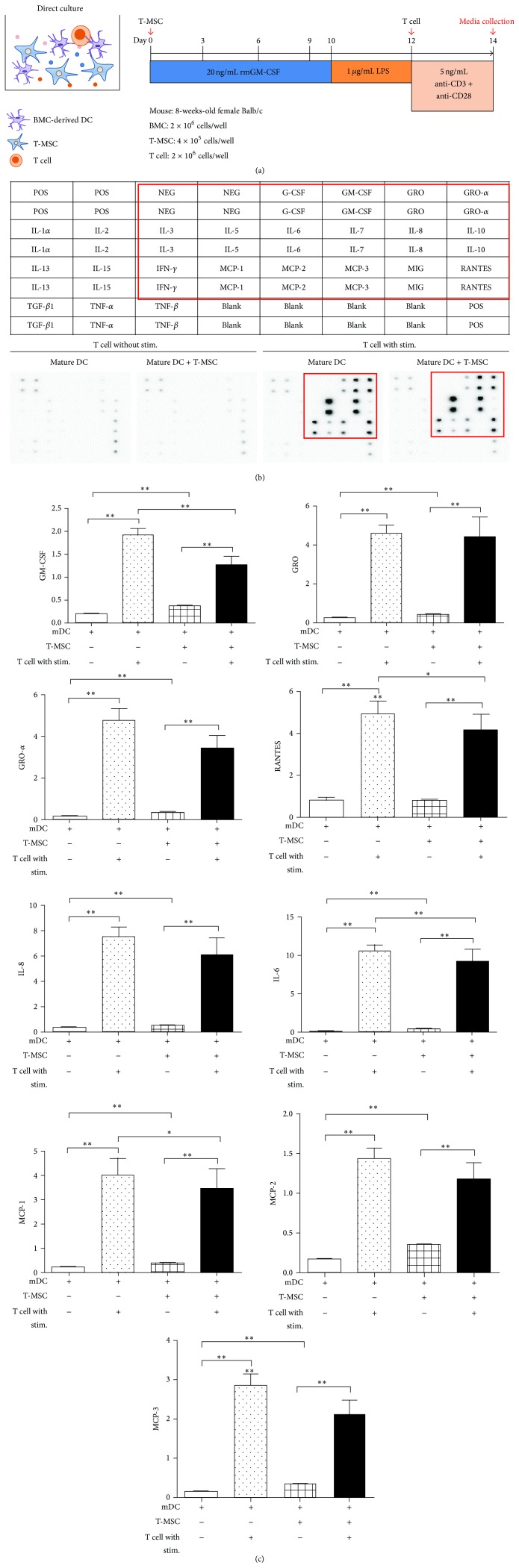
T-MSCs inhibited the release of GM-CSF, IL-6, MCP-1, and RANTES from mature DCs. (a) T cells were cocultured with mature DCs in the presence of T-MSCs. Conditioned medium was collected and subjected to Cytokine Array. (b) Plate arrangement of the Cytokine Array and cytokine spots, which show increased expression, are highlighted with a red box. (c) Expression changes were quantified and significant decreases in GM-CSF, RANTES, interleukin-6 (IL-6), and monocyte chemoattractant protein-1 (MCP-1) were observed when mature DCs and T cells were cocultured with T-MSCs.

We analyzed the BM-DC secretome upon T cell stimulation using the Cytokine Array Kit (Figure 4(a)). We found that GM-CSF, growth-regulated oncogene-*α* (GRO-*α*, also called CXCL1) regulated on activation, normal T cell expressed and secreted RANTES (also called CCL5), interleukin-8 (IL-8, also called CXCL8), IL-6, MCP-1 (also called CCL2), monocyte chemoattractant protein-2 (MCP-2, also called CCL8), and monocyte chemoattractant protein-3 (MCP-3, also called CCL7) were increased by T cell stimulation (Figure 4(b)). Among these cytokines, GM-CSF, IL-6, RANTES, and MCP-1 recruit immune cells such as DCs; these cytokines were significantly decreased upon the addition of T-MSCs. Thus, T-MSCs inhibited the secretion of inflammation-related cytokines by DCs that are normally induced by T cell stimulation (Figure 4(c)).

## 4. Discussion

In this study, we confirmed that T-MSCs inhibited the differentiation of BM-DCs induced by GM-CSF and also inhibited the maturation of BM-DCs. We found that T-MSCs suppressed the maturation of BM-derived DCs via direct contact as well as through secretion of soluble factors. Moreover, T-MSCs inhibited BM-DC-induced proliferation and differentiation of CD4^+^ T cells.

DCs play a central role in the initiation and regulation of immune responses to foreign as well as self-antigens. DCs have potent antigen presenting function and can induce CD4^+^ T cell activation and differentiation. Several DC subsets participate in various immune functions and, among these various types of DC subsets, monocyte-derived DCs, which are associated with inflammation and infection, have been actively studied [10]. When naïve CD4^+^ T cells from mouse spleens interact with mature DCs (mDCs), secretion of GM-CSF, IL-6, MCP-1, and RANTES is significantly decreased when the T cells are cocultured with T-MSC. The ability of T-MSC to inhibit RANTES secretion from T cells likely contributes to the prevention of binding between DC and T cells, which might lead to decreased immune stimulatory effect of BM-DCs. Because GM-CSF is considered a key factor for the differentiation of monocytes into inflammatory DCs [11], decreased GM-CSF under T-MSCs coculture possibly might block DC differentiation. In addition, T-MSCs inhibit differentiation of T cell subsets, which might be caused by the interruption of DC and T cell signals due to downregulated expression of class II MHC molecule expression on DC. Recently, the focus of MSC investigations has been on modulation of immune function using natural killer cells [12], T cells [13, 14], B cells [15, 16], DCs [17–19], and macrophages [20, 21] with the connection between immune cells and MSCs shown as having a major role in both innate and adaptive immunity. MSCs have been demonstrated to inhibit the maturation of monocyte-derived DCs by downregulating expression of class II MHC and costimulatory molecules [22]. The mechanism of MSC-induced inhibition of DC differentiation appears to be mediated by soluble factors, such as prostaglandin E_2_ (PGE_2_), which is secreted upon cell-to-cell contact [23]. Moreover, another mechanism used by MSCs to inhibit differentiation and maturation of DCs is production of IL-6 [24]. Prostaglandin E_2_ inhibits fungus antigen-induced interferon regulatory factor 4 (IRF4) translation [25] and IRF4 plays an important role in class II MHC expression on CD11b^+^ DCs [26]. Prostaglandin E_2_ and IL-6 produced by T-MSC may play an important role in the inhibition of MHC class II expression on DC.

There has been a focus on MSCs derived from various sources in order to avoid the ethical stigma associated with embryonic stem cells [27]. In addition, the prohibitive costs and low success rates of induced pluripotent stem cells make MSCs a more attractive prospect for immune therapy [28]. However, BM-MSCs are difficult to obtain [29] and BM yields comparatively fewer MSCs as compared to cord blood or amniotic fluid [30]. Therefore, a new source of MSCs devoid of these disadvantages is greatly needed. Hence, our group's focus has been on T-MSCs, which have the capacity to differentiate into cells of various types and did not show dominant growth in mixed-culture from multiple donors [7]. Tonsillectomy was performed about 40,000 per year in Korea and mostly bilateral palatine tonsils were removed and discarded after the surgery. On our experiment, we used one-third volume of tonsils for the preparation of mononuclear cells and we usually got 8–10 × 10^8^ cells. According to yields of cells, if we isolate mononuclear cells from bilateral tonsils from a person, we can get 6 × 10^10^ cells and this corresponds to the number of cells transplantable for a person. In this paper, we studied the role of T-MSCs in the differentiation, maturation, and antigen presentation of monocyte-derived DCs. Mouse BM-derived DCs induced by GM-GSF expressing CD11b. LPS-stimulated BM-DCs (mature DCs) showed higher expression of CD80, CD86, and class II MHC molecules compared to DCs not stimulated with LPS. However, the T-MSC-pretreated DCs showed low expression of these markers. Cocultures of DC and T-MSC inhibited DC differentiation. We investigated whether or not the inhibitory effect of T-MSCs on DC was mediated through direct contact or via secreted factor(s). To make this determination, we used a transwell barrier method, which prohibits direct contact between T-MSCs and BM-DCs. We also examined DC maturation by analyzing cell phenotypes upon coculture with T-MSCs from day 0 or day 10. We found that only T-MSCs added from day 0 inhibited DC maturation and CD4^+^ T cell proliferation. However, CD11b^+^ cell expansion and CD86 upregulation by LPS stimulation were inhibited by T-MSCs. Interestingly, CD86 and class II MHC molecules were also significantly reduced in immature DCs (i.e., without LPS stimulation). Thus, these T-MSCs exerted immune regulatory functions that are similar to MSCs derived from other sources and are mediated by soluble factors.

## 5. Conclusions

This is the first report demonstrating that T-MSCs have an immunoregulatory function: inhibition of DC maturation and differentiation via the secretome derived from T-MSCs. This finding suggests that T-MSCs possess immunosuppressive effects on DCs, which could be exploited as a cell therapy for immune-mediated disease.

## Supplementary Material

For the comparison of T-MSCs with BM-MSCs, we performed the phenotype analysis by flow cytometer and it shows that both MSCs lack of CD14, CD34, and CD45 but express CD73, CD90, and CD105 (Supplement Figure 1). Therefore, we concluded that T-MSCs, we had isolated and used in this study, show similar phenotype to that of BM-MSCs. In order to analyze the immune suppressive effect of T-MSCs on DC differentiation and maturation, we performed co-culture assay with dose response experiments in supplement Figure 2. It shows that even under 100:1 (mature DC to T-MSC ratio) decrease of CD11b, CD80, CD86 and class II MHC molecules are statistically different on DCs.
