# Corrigendum to “Immune Suppressive Effects of Tonsil-Derived Mesenchymal Stem Cells on Mouse Bone-Marrow-Derived Dendritic Cells”

**DOI:** 10.1155/2019/9071046

**Published:** 2019-03-05

**Authors:** Minhwa Park, Yu-Hee Kim, Jung-Hwa Ryu, So-Youn Woo, Kyung-Ha Ryu

**Affiliations:** ^1^Department of Microbiology, School of Medicine, Ewha Womans University, Seoul 158-710, Republic of Korea; ^2^Department of Pediatrics, School of Medicine, Ewha Womans University, Seoul 158-710, Republic of Korea

In the article titled “Immune Suppressive Effects of Tonsil-Derived Mesenchymal Stem Cells on Mouse Bone-Marrow-Derived Dendritic Cells” [1], the funding of the Intramural Research Promotion Grants from Ewha Womans University School of Medicine was missing from the Acknowledgments section. Therefore, the section should be updated as follows:

This work was supported by the Basic Science Research Program through the National Research Foundation of Korea, which was funded by the Ministry of Education, Science, and Technology (NRF-2012M3A9C6049823) and the Intramural Research Promotion Grants from Ewha Womans University School of Medicine.
