# Supplementary material for: Immune Suppressive Effects of Tonsil-Derived Mesenchymal Stem Cells on Mouse Bone-Marrow-Derived Dendritic Cells
Source: Stem Cells Int. 2015 Feb 16;2015:106540. doi: 10.1155/2015/106540 (PMC4345276; doi:10.1155/2015/106540)
Supplement: Supplementary file 1 — For the comparison of T-MSCs with BM-MSCs, we performed the phenotype analysis by flow cytometer and it shows that both MSCs lack of CD14, CD34, and CD45 but express CD73, CD90, and CD105 (Supplement Figure 1). Therefore, we concluded that T-MSCs, we had isolated and used in this study, show similar phenotype to that of BM-MSCs. In order to analyze the immune suppressive effect of T-MSCs on DC differentiation and maturation, we performed co-culture assay with dose response experiments in supplement Figure 2. It shows that even under 100:1 (mature DC to T-MSC ratio) decrease of CD11b, CD80, CD86 and class II MHC molecules are statistically different on DCs. [file 106540.f1.pdf]

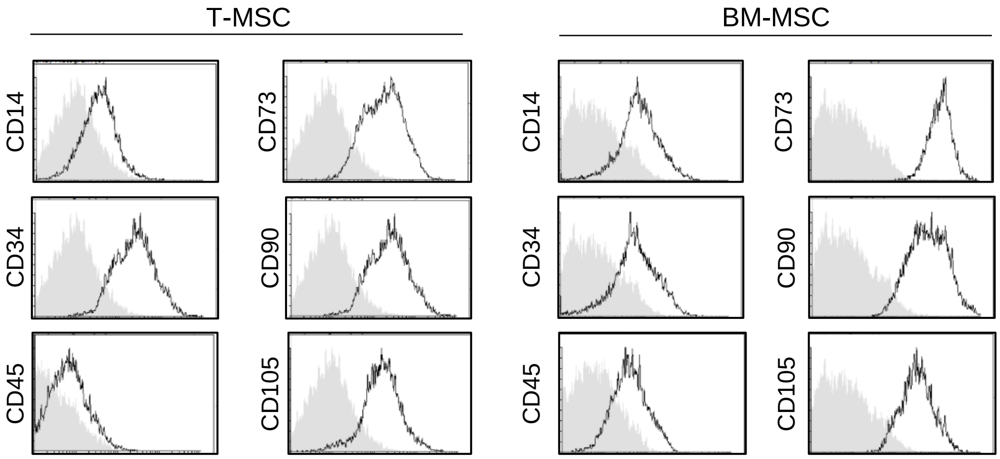

Figure 1. Analysis of phenotype for in vitro cultured T-MSCs and BM-MSCs. T-MSCs and BM-MSCs were cultured in 10% FBS containing DMEM media for 7 days under 37℃, 5% CO_2 incubator. Cells were collected and stained for flow cytometer analysis. Hematopoietic cell marker (CD14, CD34, and CD45) and mesenchymal stem cell marker (CD74, CD90, CD105) were measured by flow cytometer. Phenotype of T-MSCs and BM-MSC were seen negative expression of CD14, CD34, CD45 and positive expression of CD73, CD90, CD105 (black lines). Grey area represent unstained cells. Antibodies used: anti-human CD14 (M5E2, mouse IgG_{2a}, BD), anti-human CD34 (581, mouse IgG_1, BD), anti-human CD45 (2D1, mouse IgG, BD), anti-human CD73 (AD2, mouse IgG_1, BD), anti-human CD90 (5E10, mouse IgG , BD), and anti-human CD105 (43A3, mouse IgG_1, Biolegend)

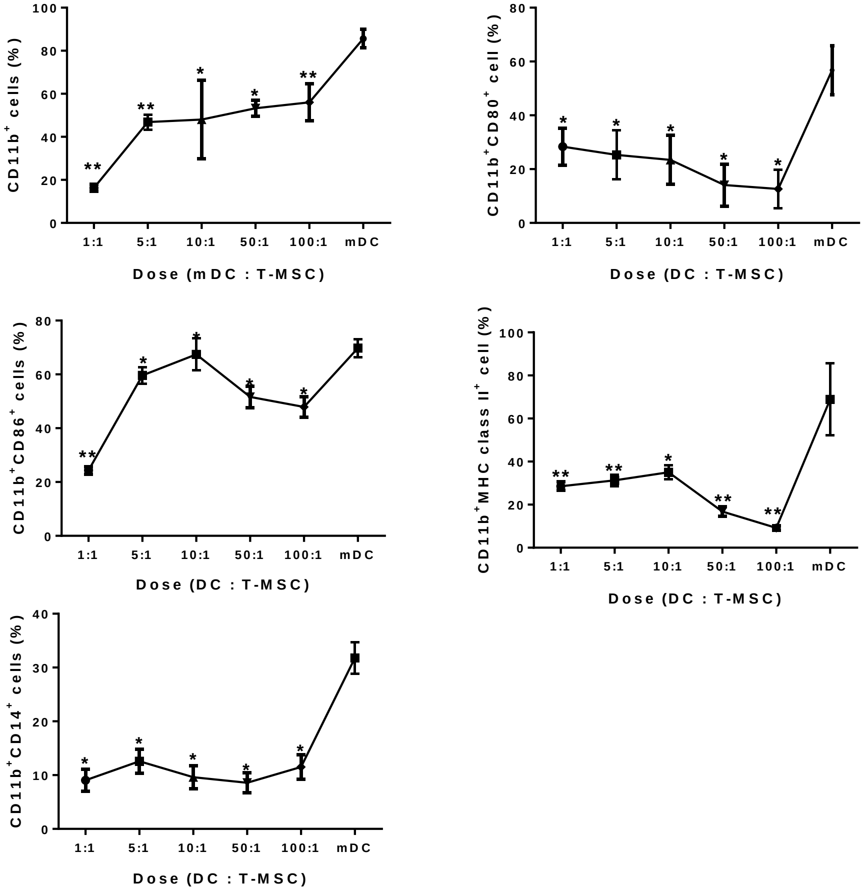

Figure 2. T-MSCs were effected by variety DCs dose. BMCs were induced imDCs under RPMI 1640 media add 20 ug/ml GM-CSF for 10 days. LPS stimulated imDCs for maturation, after 48 hours, cell were collected and stained for surface marker as CD11b, CD80, CD86, MHC class II, and CD14. Statistics of positive cells percentage was analyzed between mDCs vs T-MSCs-treated mDCs (*, $P<0.05$ and **, $P<0.001$).
